# Impaction of the Colon in a Horse

**Published:** 1882-07

**Authors:** 


### VIII.—IMPACTION OF THE COLON IN A HORSE.

REPORTED BY DRS. WALTON AND M'LELLAN.

In April, 1882, a horse was brought to the hospital with the history that he had been comparatively idle for three months. At the end of this time he was put to steady work on a cart. After two days of hard work his appetite failed, and there was no discharge ot fæcal matter.

The owner, however, continued to work the animal a day longer, having administered a dose of oleum lini the night previous. When brought to the hospital there had been no evacuation from the bowels for the previous seven days, nor had he taken any food worthy of note.

The animal was evidently suffering considerable pain. A diagnosis of impaction of the colon was made, and an unfavorable prognosis advanced. Oleum lini, with oleum tiglii, was given. The animal, however, died on the eighth day of the disease.

Necropsy the following morning. At one point the colon was completely filled with hardened fæcal matter, which was forty-one centimetres long. Just anterior to the occlusion the colon had given way, which was the probable cause of the sudden death.

COLUMBIA VETERINARY COLLEGE HOSPITAL, NEW YORK CITY, 1882.

